# Statistical and Ontological Analysis of Adverse Events Associated with Monovalent and Combination Vaccines against Hepatitis A and B Diseases

**DOI:** 10.1038/srep34318

**Published:** 2016-10-03

**Authors:** Jiangan Xie, Lili Zhao, Shangbo Zhou, Yongqun He

**Affiliations:** 1 Key Laboratory of Dependable Service Computing in Cyber Physical Society, Ministry of Education, Chongqing University, Chongqing, 400044, China; 2 University of Michigan Medical School, Ann Arbor, Michigan, 48109, USA; 3 Department of Biostatistics, University of Michigan, Ann Arbor, Michigan, 48109, USA

## Abstract

Vaccinations often induce various adverse events (AEs), and sometimes serious AEs (SAEs). While many vaccines are used in combination, the effects of vaccine-vaccine interactions (VVIs) on vaccine AEs are rarely studied. In this study, AE profiles induced by hepatitis A vaccine (Havrix), hepatitis B vaccine (Engerix-B), and hepatitis A and B combination vaccine (Twinrix) were studied using the VAERS data. From May 2001 to January 2015, VAERS recorded 941, 3,885, and 1,624 AE case reports where patients aged at least 18 years old were vaccinated with only Havrix, Engerix-B, and Twinrix, respectively. Using these data, our statistical analysis identified 46, 69, and 82 AEs significantly associated with Havrix, Engerix-B, and Twinrix, respectively. Based on the Ontology of Adverse Events (OAE) hierarchical classification, these AEs were enriched in the AEs related to behavioral and neurological conditions, immune system, and investigation results. Twenty-nine AEs were classified as SAEs and mainly related to immune conditions. Using a logistic regression model accompanied with MCMC sampling, 13 AEs (*e.g.*, hepatosplenomegaly) were identified to result from VVI synergistic effects. Classifications of these 13 AEs using OAE and MedDRA hierarchies confirmed the advantages of the OAE-based method over MedDRA in AE term hierarchical analysis.

Vaccine pharmacovigilance is the pharmacological science relating to the detection, assessment, understanding, communications, and prevention of adverse events (AEs) following vaccine immunization. As one of the greatest inventions in modern medicine, vaccine has contributed greatly to the amelioration of human misery and the increase in life expectancy in the past two centuries. For example, smallpox, a fatal infectious disease, has been eradicated owing to vaccination^1^. Other disabling and lethal diseases, like poliomyelitis and measles, are currently targeted for eradication^2^,^3^. However, vaccinations are often accompanied with AEs, some of which may be serious and even fatal^4^. Understanding the vaccine AE (VAE) profiles is crucial to predict potential serious AEs (SAEs) and improve vaccine safety.

At present, most VAE research focuses on reporting and analysis of AEs associated with single vaccines. Considering that most drug-drug interactions (DDIs) may account for up to 30% of unexpected adverse drug events^5^,^6^, there are likely interactions between different vaccines administered to an individual at the same time. Nowadays, the combination vaccines become more and more common since vaccinees obtain the same protection with one vaccination as they do from individual vaccines given separately, which result in fewer shots and less cost^7^. Six combination vaccines (*i.e.*, Comvax, Twinrix, Pediarix, ProQuad, Kinrix, and Pentacel) have been approved by the Food and Drug Administration (FDA) to use in the USA^8^. As researchers develop new vaccines against more diseases, more combination vaccines may become available in the future. Even though these combination vaccines are considered highly safe, some potential AEs, including many SAEs, may be induced by the interactions among monovalent vaccines when they are administered together. Therefore, it is important to generate accurate and early prediction of vaccine-vaccine interactions (VVIs) to prevent the VVI-induced newfound or serious AEs. Unfortunately, rare studies have paid attention to VVIs.

In order to monitor post-licensure vaccine AEs, the Centers for Disease Control and Prevention (CDC) and the FDA established the Vaccine Adverse Event Report System (VAERS) surveillance program in 1990^9^. VAERS typically receives over 30,000 case reports annually from various submitters including professional healthcare providers, vaccinees, vaccinees’ relatives, and vaccine manufacturers. However, as a passive surveillance system, VAERS is subject to a number of well-described limitations such as high variability in report quality, biased reporting, underreporting and the inability in VAE causality determination^10^. Nevertheless, with statistical support, VAERS has been used widely to identify potential vaccine safety issues^4^,^11^,^12^.

In VAERS, each case report is annotated by certified coders who manually assign individual AEs with the codes of the Medical Dictionary for Regulatory Activities (MedDRA), a standard vocabulary nomenclature^13^. Until now, there are 9,893 MedDRA terms used in VAERS. While MedDRA has played a central role in standardizing vocabulary in the scope of AE reporting, there exist several issues related to the MedDRA usage^14^. First, MedDRA does not provide any term definitions, which may cause confusion. Second, MedDRA has a poorly defined hierarchical structure, making it difficult to use for advanced AE clustering analysis. To improve the AE representation and organization, the community-based Ontology of Adverse Events (OAE) has recently been developed^15^. OAE organizes AE terms in a logical hierarchy based on pathological processes of AE symptoms. In OAE, an AE term denotes a pathological bodily process in a patient that occurs after a medical intervention (*e.g.*, vaccination). Since MedDRA is the standard method for AE representation in VAERS, OAE terms have been mapped to corresponding MedDRA terms to support VAERS AE classification. After the MedDRA-OAE term mapping, the VAERS contents associated with the MedDRA terminology can be analyzed by an OAE-based AE classification method. Compared to MedDRA, OAE has been empirically showed to have superior performance in classifying different AEs associated with live attenuated or killed inactivated influenza vaccines^11^.

Hepatitis A and B are vaccine-preventable epidemic diseases that are caused by highly contagious hepatitis A and B viruses, respectively. The CDC Advisory Committee on Immunization Practices (ACIP) recommends hepatitis A and B vaccinations for all children at age 1 year^16^. For adults, especially healthcare workers, patients with chronic liver disease, injection drug users, prisoners, travelers to endemic areas, and men who have sex with men should also get the vaccines timely^17^. Meanwhile, for the use of the hepatitis A and B vaccines, it is recommended to pay attentions to the risk of post-vaccination AEs, particularly those SAEs such as autoimmune diseases (*e.g.*, multiple sclerosis, arthritis, and myelitis) that have been postulated to be caused by hepatitis vaccines^12^.

GlaxoSmithKline Biologicals manufactures three vaccines, Havrix, Engerix-B, and Twinrix, against hepatitis A and B diseases. Havrix is a monovalent vaccine that contains 1,440 ELISA units (EL.U) of the inactivated hepatitis A virus. Engerix-B is a monovalent vaccine containing 20 μg of recombinant protein hepatitis B surface antigen (HbsAg). Twinrix is a combination vaccine that is a mixture of the Havrix (half dose) and Engerix-B (full dose), containing 720 EL.U of inactivated hepatitis A virus and 20 μg of recombinant HbsAg^18^. Given that the three licensed vaccines are all manufactured by the same company and the formulation of Twinrix is a combination vaccine out of the mixture of Havrix and Engerix-B, the investigation of the responses stimulated by the three vaccines provides an ideal use case of VVI studies. Clinical studies have indicated that Twinrix is well tolerated and displays no increased reactogenicity compared to monovalent vaccines Havrix and Engerix-B administered concurrently^18^,^19^,^20^. However, it was found that the titers of antibodies to hepatitis A and hepatitis B viruses were significantly higher after administration of the combined or mixed vaccines than after separate injections of the monovalent vaccines^19^,^20^, suggesting a potential interaction between the two monovalent vaccines. The up-regulated antibody titers were likely due to the increased local production of cytokines and consequent enhancement of macrophage activity that result from the interactions between the components of Havrix and Engerix-B^21^. Nowadays, these monovalent and combination hepatitis A and B vaccines have been used in market more than ten years, and there are many AE case reports associated with these vaccines recorded in VAERS. However, systematic comparison and analysis the AE profiles of these monovalent and combination hepatitis A and B vaccines with large scale post-licensure AE case report data has not been performed yet. The analysis of VAERS AE data related to Havrix, Engerix-B, and Twinrix provides us an ideal case to study the AEs associated with hepatitis A and B vaccines and their combination vaccine.

In this study, we investigated the comparative profiles of AEs associated with Havrix, Engerix-B, and Twinrix. The statistically significant AEs were then classified and analyzed using OAE-based methods. Given Twinrix being a combination vaccine composed of Havrix and Engerix-B, we hypothesized that some Twinrix-associated AEs would be induced by the interaction between Havrix and Engerix-B. To address this hypothesis, we designed and implemented a novel statistical method that combines a logistic regression modeling method with a Markov Chain Monte Carlo (MCMC) sampling^22^.

## Methods

### Adverse event data extraction

Vaccinees are often administered with more than one vaccine during a short period of time. In this case, it is impossible to associate following AEs with individual vaccines. To improve the accuracy of predicting vaccine-specific AEs, especially AEs associated with the interaction between Havrix and Engerix-B, we only extracted the AE case reports from those VAERS cases where the vaccinees were administered with only one hepatitis vaccine (*i.e.*, Havrix, Engerix-B, or Twinrix).

Our study considered the effects of different variables. Twinrix was approved for use in the USA in persons 18 years of age or older starting on May, 11, 2001^23^. In comparison, Havrix and Engerix-B had already been used for all ages before 2001. To ensure comparability among the VAEs of these three vaccines, we included only VAERS AE reports received from May 2001 to January 2015 for those vaccinees who were aged at least 18 years old.

The VAERS cases filtered based on the above three criteria (age, reporting time, and only one vaccine administered) were used for the following statistical analysis (*e.g.*, PRR) to identify statistically significant AEs. Related AE case report details were extracted from the CDC Wonder VAERS data search website (http://wonder.cdc.gov/controller/datarequest/D8).

### Statistical data analysis with PRR, Chi-square (*x* ^2^) test, and base level filtration

The proportional reporting ratio (PRR)^24^ is a main statistical method used in our data analysis. Basically, the VAERS database can be viewed as a contingency table with rows representing the MedDRA coding terms and columns representing the vaccine products. Each cell in the table contains a value that gives the number of reports for the AE of interest and the vaccine of interest. Using the contingency table data structure, PRR calculates the proportion of a specific AE for a vaccine of interest where the comparator is all other vaccines in the VAERS database^24^. As described above, the restriction of vaccinee age (18 years of age or older), case reporting period (May 2001 to January 2015), and single vaccine administration was applied in our data retrieval from the VAERS database. A large PRR score of a specific vaccine AE indicates that the AE has been disproportionately reported for that vaccine, compared with all the other vaccines. We applied the standard PRR score cutoff of 2, *i.e.*, only those AEs with PRR ≥ 2 were further considered^24^,^25^.

In parallel with the PRR signal detection, we also applied a *x*^2^ test to statistically analyze the likelihood of individual AE terms associated with specific vaccines^26^. The *x*^2^ test calculation also relies on the contingency table described above. An AE is called significant when its *x*^2^ score is greater than 4, which is equivalent to a *p*-value of approximately 0.05 or smaller^11^. More details about how to compute the scores of PRR and *x*^2^ for each AE in each group based on a 2 × 2 contingency table is provided in Supplementary Table S1.

To filter out background noises effectively, when the total case report number is less than 1,500, we also applied a minimal sample size cutoff of 3 case reports as a base level filtration for each AE to be further considered. When the total case report number is greater than 1,500, the cutoff was set to be 0.2% of total reports for each AE. Such a base level filtration means that at least 2 out of 1,000 cases should report the AE of interest^11^,^27^. The combined use of PRR and a minimal sample size cutoff is also called screened PRR method (SPRR)^25^.

Note that the MedDRA controlled terminology system used by VAERS contains many terms indicating laboratory test result *normal* or *negative (e.g.*, ‘*blood albumin normal*’ and ‘*hepatitis B test negative*’). These normal or negative laboratory test results are not involved in any AEs. In addition, many MedDRA terms, such as ‘*lymphocyte percentage*’, ‘*rheumatoid factor*’, ‘*CSF cell count*’ and ‘*electroneurography*’ only represent plain variables or plain test methods and are actually not AEs. Such ambiguous and non-informative MedDRA terms were removed in our AE analysis.

### OAE-based vaccine adverse event classification

The hierarchical structures of AE terms in OAE and MedDRA are largely different. For better comparison and analysis, statistically significant MedDRA terms associated with Havrix, Engerix-B, and Twinrix were mapped to corresponding OAE terms. Through MedDRA-OAE term mapping, the AE terms were classified and analyzed using OAE and MedDRA hierarchical structures^11^. The OntoFox software program was used to automatically extract Havrix-, Engerix-, and Twinrix-specific AE terms, their parent terms, and associated hierarchies in OAE^28^. Note that the OAE version 1.1.316 was used in this study and the whole hierarchical structure of OAE can be viewed in Ontobee (http://www.ontobee.org/ontology/OAE)^29^. The MedDRA hierarchies of selected AE terms were extracted from the MedDRA version obtained in the BioPortal website (http://bioportal.bioontology.org/ontologies/MEDDRA) on December 22, 2015. The hierarchical results were all visualized and compared using the Protégé-OWL editor^30^.

### SAE classification

According to FDA, an AE is any undesirable experience associated with the use of a medical product in a patient, and a SAE was defined as an AE at any dose that: (i) results in death, (ii) is life-threatening, (iii) results in hospitalization, (iv) results in disability or permanent damage, (v) is congenital anomaly or birth defect, (vi) requires intervention to prevent permanent impairment or damage, or other serious medical events^31^. In this paper, for AEs associated with the hepatitis vaccines, we classified an AE as a SAE based on the FDA’s definition as well as the existing classification in previous publications^4^,^11^,^12^,^32^,^33^.

### Identification of VVIs by developing and applying a new logistic regression model accompanied with MCMC sampling

To analyze the vaccine-vaccine interactions (VVIs), we developed and applied a VVI-specific logistic regression model. In this method, we modeled the probability of an AE (present/absent) as a function of two vaccines:


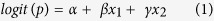


In the above logistic regression, *p* denotes the probability of AE, and (*x*_1_, *x*_2_) is the design vector for the two vaccines. For example, (1, 0) is for a case to have hepatitis A vaccine, (0, 1) is for hepatitis B vaccine, and (0, 0) is for hepatitis AB combination vaccine. We adopted Markov Chain Monte Carlo (MCMC) sampling to obtain posterior distributions of the model parameters (α, β, γ) using a MCMClogit function in *R*^22^,^34^. Then these parameters (or their functions) were used to estimate probabilities of AEs. Specifically, the probability of AE for hepatitis A (*p*_*A*_), hepatitis B (*p*_*B*_), and hepatitis AB (*p*_*AB*_) are shown in Equation (2~4).


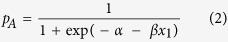



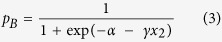



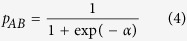


For a particular AE, the synergistic effect of vaccine A and B is defined as the probability of an AE for the vaccine combination that is larger than the simple sum of AE probabilities of the two vaccines alone^35^,^36^. That is, the vaccine A and B has synergistic effect if the fold change (FC) 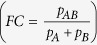 is large. Specifically, we calculated two posterior probabilities: *p*_*FC*2_ = *P*(*FC* > 2) and *p*_*FC*1_ = *P*(*FC* < 1). The strong synergistic effect is evidenced by a large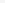 *p*_*FC*2_ and a small *p*_*FC*1_. In this study, we selected AE with a significant synergistic effect for hepatitis A and B vaccines if *p*_*FC*2_ > 0.80 and *p*_*FC*1_ < 0.05.

## Results

**Figure 1:**
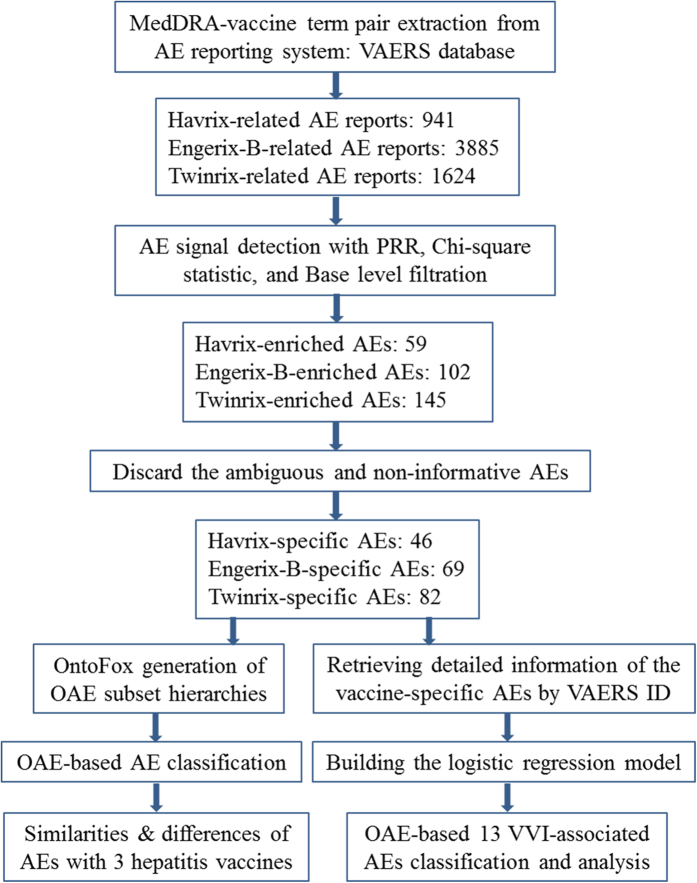
Overall project workflow and related results.

The general project workflow shown in Fig. 1 outlines different steps in our study and the results out of each step. The details of these analysis processes are provided below.

### Extracting AEs associated with Havrix, Engerix-B and Twinrix from VAERS

As described in the Methods section, we retrieved and analyzed the VAE cases reported since May 2001 due to the fact that all the three vaccines were on the market after that time. From May 2001 through January 2015, in vaccinees 18 years of age or older, the VAERS database contained information of 161,446 AE reports for 204 vaccine products. With this VAERS dataset, 941, 3,885, and 1,624 AE case reports are specifically related to the vaccination of Havrix, Engerix-B, and Twinrix, respectively (Fig. 1). From these case reports, we identified 1,093, 2,118, and 1,851 AEs (coded with MedDRA terms) specifically associated with Havrix, Engerix-B, and Twinrix, respectively. Note that in these reported cases, the vaccinees were inoculated with only one hepatitis vaccine (*i.e.*, Havrix, Engerix-B or Twinrix), and no other vaccines were co-administered.

### Statistically significant VAEs identified

**Figure 2:**
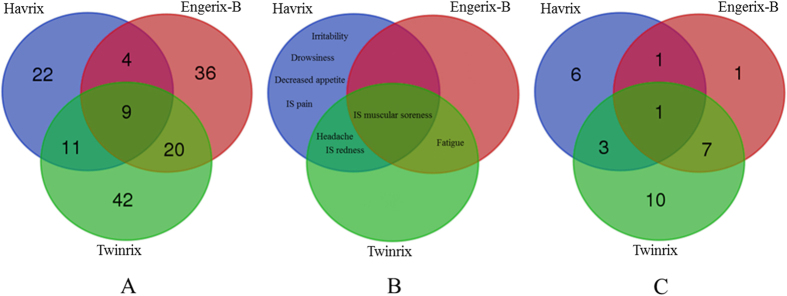
Venn diagram summary of Havrix-, Engerix-B-, and Twinrix-specific AEs. (**A**) Statistically significant AEs associated with the three vaccines as identified from VAERS data analysis. (**B**) OVAE-recorded AEs known to be associated with the three vaccines. IS: injection site. (**C**) SAEs associated with these three vaccines based on VAERS data analysis.

By adopting the screening criteria include PRR score (≥2), Chi-square score (≥4), and the number of reports (*i.e.*, ≥3 for Havrix, ≥8 for Engerix-B, and ≥4 for Twinrix), our study identified 59, 102, and 145 AEs significantly associated with Havrix, Engerix-B, and Twinrix, respectively. Among these AE terms, 13 AEs associated with Havrix (*e.g.*, ‘blood albumin normal’ and ‘hepatitis A antibody’), 33 AEs with Engerix-B (*e.g.*, ‘HIV test negative’ and ‘electromyogram normal’), and 63 AEs with Twinrix (*e.g.*, ‘lymphocyte percentage’, ‘immunoglobulins’ and ‘CSF lactate normal’) are ambiguous, non-informative, and/or indeed not AEs. These terms were discarded for the following analysis. Eventually, 46 Havrix-specific AEs (Table 1), 69 Engerix-B-specific AEs (Table 2), and 82 Twinrix-specific AEs (Table 3) remained (Fig. 1). Based on a Venn diagram analysis (Fig. 2A), 9 AE symptoms (*i.e.*, hepatitis, liver disorder, gamma-glutamyltransferase level increased, transaminases level increased, jaundice, blood bilirubin level increased, diplopia, paresis, and vasculitis) were shared by all three vaccines. In addition, Engerix-B and Twinrix shared 20 AE symptoms (*i.e.*, central nervous system inflammation, central nervous system lesion, systemic lupus erythematosus, multiple sclerosis, muscle atrophy, vertigo, dysaesthesia, formication, proteinuria, myelitis, demyelination, visual disturbance, optic neuritis, hepatitis B, rheumatoid arthritis, psoriasis, polyneuropathy, muscle disorder, sensory disturbance, and hepatomegaly). Havrix and Engerix-B shared 4 AE symptoms (*i.e.*, osteoarthritis, fibrosis tendinous, myofascitis, and fasciitis). Havrix and Twinrix shared 11 AE symptoms (*i.e.*, ocular icterus, anaphylactic shock, aspartate aminotransferase level increased, chromaturia, abortion, pelvic pain, hepatitis A, vaginal haemorrhage, impaired work ability, alanine aminotransferase level increased, hepatic enzyme increased). In total, 144 unique statistically significant AEs were identified to be associated with at least one of these there vaccines.

While VAERS reported AEs came from spontaneous case reports, the AEs listed in the official FDA vaccine package insert documents were generated from randomized, well-controlled clinical trials^37^,^38^,^39^. The AEs specific for individual vaccines recorded in the FDA package insert documents have been represented and classified in the Ontology of Vaccine Adverse Events (OVAE)^40^. To compare the AEs generated from the VAERS data and the FDA package insert knowledge, AEs related to the three vaccines represented in the OVAE were also extracted and compared (Fig. 2B). In OVAE, there are 7 AE types associated with Havrix, 4 AEs associated with Twinrix, and 2 AEs associated with Engerix-B (Fig. 2B). Among these AEs, two AE symptoms (*i.e.*, headache and injection site redness) are shared between Havrix and Twinrix, one AE symptom (*i.e.*, fatigue) shared between Engerix-B and Twinrix, and one AE symptom (*i.e.*, injection site muscular soreness) shared among all three vaccines. Overall, compared to the VAERS results, there are less numbers of AEs recorded in FDA package inserts and represented in OVAE, and FDA-recorded AEs associated with these three vaccines are in general mild and self-limited.

### AE hierarchical classification based on the OAE method

After statistically significant AEs were detected, we analyzed these AEs using an OAE-based classification method. Tables 1, 2, 3, [Table t2], [Table t3] summarizes the Havrix-, Engerix-B-, and Twinrix-specific AEs after the OAE clustering analysis, respectively. Supplementary Figs S1–S3 records the OAE hierarchies of statistically significant AEs associated with these three vaccines. As shown in Tables 1, 2, 3, [Table t2], [Table t3] and Supplementary Figs S1–S3, the most frequently identified AE category is the behavioral and neurological AE category that includes 29 unique AEs for all three vaccines. The other commonly identified categories include immune system AEs and investigation result abnormal AEs. Specifically, for Havrix (Table 1), the AEs included in pregnancy, neonatal or perinatal disorder (*e.g.*, abortion spontaneous and unintended pregnancy) were relatively frequent AEs. Engerix-B was associated with many nervous system AEs (*e.g.*, demyelination, hyperreflexia, and optic neuritis), eye disorders (*e.g.*, visual acuity reduced, double vision, and visual disturbance), and musculoskeletal or connective tissue AEs (*e.g.*, fasciitis, fibrosis tendinous, and myofascitis) (Table 2). For the combination vaccine Twinrix (Table 3), many commonly identified AEs occurred at the hepatobiliary system (*e.g.*, jaundice and liver disorder), cardiovascular system (*e.g.*, circulatory collapse and cardiovascular disorder), and nervous system (*e.g.*, myelitis, optic neuritis, and polyneuropathy). It is remarkable that the number of AEs associated with Twinrix was more than that with Havrix or Engerix-B.

### SAEs associated with hepatitis A and B vaccines

**Figure 3:**
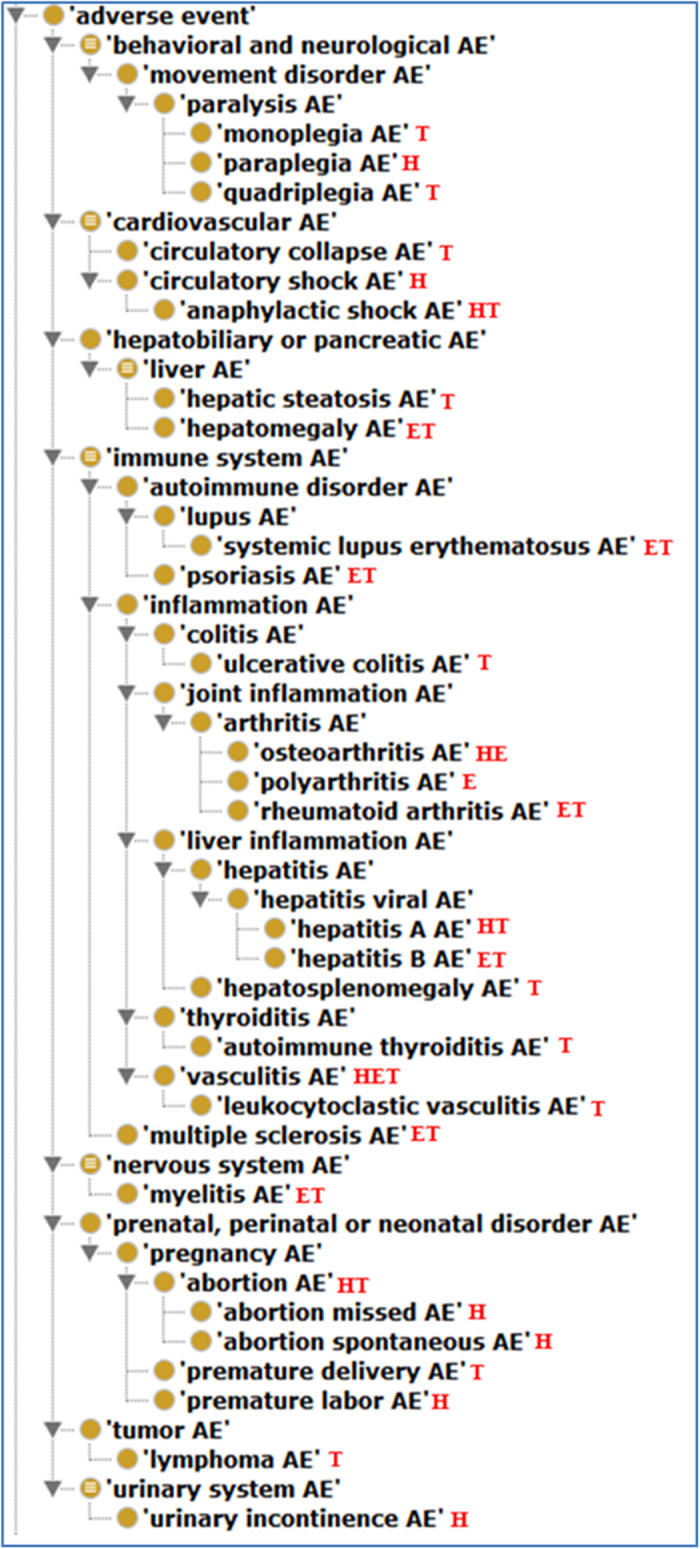
Classification of 29 SAEs associated with three vaccines using OAE. **H** represents a SAE associated with Havrix. **E** represents a SAE associated with Engerix-B. **T** represents a SAE associated with Twinrix. **HE** represents a SAE shared by Havrix and Engerix-B. **HT** represents a SAE shared by Havrix and Twinrix. **ET** represents a SAE shared by Engerix-B and Twinrix. **HET** represents a SAE shared among all three vaccines.

Among all statistically significant VAEs, our analysis identified 11, 10, and 21 SAEs associated with Havrix, Engerix-B, and Twinrix, respectively (Tables 1, 2, 3, [Table t2], [Table t3]). A Venn diagram analysis of these vaccine-specific SAEs identified 29 unique SAEs (Fig. 2C). Among these 29 SAEs, only vasculitis was shared among all three vaccine groups. Havrix and Engerix-B were both associated with osteoarthritis AE. Havrix and Twinrix shared the abortion, anaphylactic shock, and hepatitis A AEs. Seven SAE symptoms (*i.e.*, systemic lupus erythematosus, multiple sclerosis, myelitis, hepatitis B, rheumatoid arthritis, psoriasis, and hepatomegaly) were shared by Engerix-B and Twinrix. Based on the OAE classification (Fig. 3), these SAEs (13 out of 29 SAEs) are mainly distributed in the immune system. Remarkably, among the 13 immune system SAEs, 8 SAEs (*i.e.*, multiple sclerosis, rheumatoid arthritis, polyarthritis, myelitis, autoimmune thyroiditis, psoriasis, ulcerative colitis, and systemic lupus erythematosus) are autoimmune-related disorders.

### Thirteen AEs identified out of synergistic VVIs between Havrix and Engerix-B

In this study, we developed a new statistical method by combining the established logistic regression model with MCMC sampling to identify the hidden interactions among the hepatitis vaccines. For 144 unique statistically significant AEs associated with Havrix, Engerix-B, and Twinrix, 13 AEs were satisfied with the defined threshold (*p*_*FC*2_ > 0.80 and *p*_*FC*1_ < 0.05) (Table 4). As shown in Table 4, for Havrix, hepatic steatosis was reported only once, and the other 12 AEs were not reported. For Engerix-B, three AEs (*i.e.*, hepatosplenomegaly, premature delivery, and sinus tachycardia) were not reported, and the other 10 AEs were reported but were not within the list of statistically significant AEs (Table 2). However, for Twinrix, each of the 13 AEs was reported for at least 4 times (Table 4), and these 13 AEs were all statistically significantly associated with Twinrix (Table 3). Our statistical analysis results further indicate that there is a high probability that these Twinrix-associated AEs are out of a synergistic interaction between the components of Havrix and Engerix-B.

**Figure 4:**
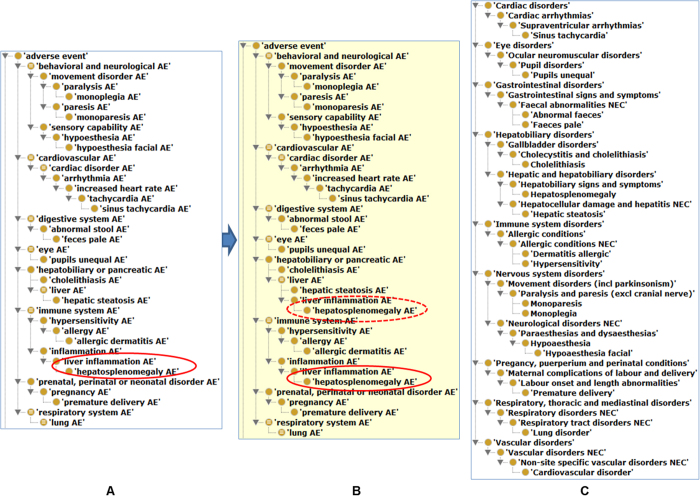
Hierarchical structures of 13 AEs associated with VVI as classified based on OAE and MedDRA. (**A**) Asserted OAE hierarchy of the 13 AEs and related top level classes. (**B**) Inferred OAE hierarchy. The ELK reasoner (version 0.4.10) was used for reasoning. After the reasoning, ‘liver inflammation AE’, highlighted by a dotted red oval, was inferred to be a ‘liver AE’. See more detail on the reasoning in the text. (**C**) MedDRA hierarchy of the 13 AEs and related top level classes. The Protégé-OWL editor was used to display the hierarchies, perform the reasoning, and generate the screenshots.

Among the 13 AEs (Table 4), hepatosplenomegaly, premature delivery, and hepatic steatosis belong to SAEs. Based on the OAE classification, the 13 AEs associated with VVIs are mainly involved in behavioral and neurological conditions (*i.e.*, monoparesis, monoplegia, and hypoesthesia facial), immune system (*i.e.*, allergic dermatitis and hepatosplenomegaly) and hepatobiliary condition (*i.e.*, hepatic steatosis, and cholelithiasis) (Fig. 4A,B). These enriched AE categories are similar to the patterns found in the AE and SAE classification (Supplementary Figs S1–S3 and Fig. 3).

### Confirmation of the OAE advantaged over MedDRA in AE classification

A previous empirical study found that OAE provided better AE classification than MedDRA in AE classification^11^. To further confirm the results and illustrate the differences between OAE and MedDRA in their applications in AE classification, we applied both OAE and MedDRA to identify the hierarchical structures of the 13 VVI-associated AEs described above (Fig. 4). Many AE terms may be classified under two or more parent terms. For example, ‘liver inflammation AE’ is fit under ‘inflammation AE’ or ‘liver AE’ (Fig. 4A,B). The approach of asserting more than one parent terms in ontology is called multiple inheritance, which often makes an ontology difficult to manually maintain and update^41^. To avoid multiple inheritances, OAE asserts only one parent term, and allows the other parent term(s) to be obtained automatically by reasoning^15^. In the above example, the ‘liver inflammation AE’ was asserted as a subclass of ‘inflammation AE’. After reasoning (based on internal logical axiom definitions), ‘liver inflammation AE’ was inferred to be a ‘liver AE’ as well (Fig. 4B). Such a feature does not exist in MedDRA.

Another difference in OAE and MedDRA exists in their strategies for basic hierarchical construction. As shown in Fig. 4C, MedDRA includes many terms ended with “NEC” (*i.e.*, “not elsewhere classified”), for example, ‘faecal abnormalities NEC’, ‘neurological disorders NEC’, and ‘respiratory disorders NEC’. Such an “NEC” term definition style is arbitrary and ambiguous, often leading to confusion and unclear classification results. For instance, the parent MedDRA term of ‘abnormal feces’ is ‘faecal abnormalities NEC’, which is confusing and logically incorrect. In addition, MedDRA misses obvious parent-child term logic. For example, MedDRA classifies ‘feces pale’ and ‘abnormal feces’, or ‘dermatitis allergic’ and ‘hypersensitivity’ in the same hierarchical levels (Fig. 4C). These are logically incorrect since in reality, a ‘feces pale’ is a subclass of ‘abnormal feces’, and ‘dermatitis allergic’ is a subclass of ‘hypersensitivity’ (Fig. 4A).

In summary, this comparative study further confirmed that OAE provides better classification outcomes than MedDRA. Since MedDRA is the default AE reporting terminology in VAERS, our approach of MedDRA-OAE term mapping followed by OAE hierarchy classification proved to be a valid method in VAERS AE studies.

## Discussion

To the best of our knowledge, current study is the first to compare and analyze the AEs associated with hepatitis A vaccine Havrix, hepatitis B vaccine Engerix-B, and hepatitis A/B combination vaccine Twinrix. Our statistical and ontological analyses of the VAERS data found that the three hepatitis vaccines were associated with 144 unique AEs (including 29 SAEs), mainly occurring in behavioral and neurological, immune, and investigation result abnormal. We also analyzed the VVIs between the hepatitis A and B vaccines by developing and implementing a new method of logistic regression modeling accompanied with MCMC sampling. Our VVI analysis identified 13 AEs out of the synergistic interaction between hepatitis A and B vaccines. These VVI-associated AEs were mainly involved in behavioral and neurological, immune, and hepatobiliary conditions.

Since the VAERS passive surveillance system has many limitations including underreporting, incomplete information in many reports, and lack of a direct and unbiased comparison group^9^,^10^,^42^, direct and naïve usages of the VAERS data may result in wrong assertions of causal relations between vaccines and AEs. Nevertheless, the combinative usage of bioinformatics and statistical methods (*e.g.*, PRR and Chi-square test) to retrieve and analyse the VAERS data can still generate many meaningful and interpretable results and draw sensible hypotheses between vaccines and AEs. For example, the research based on VAERS data by Sirarat *et al.* suggested that the live attenuated influenza vaccine (LAIV) had lower chance of inducing Guillain-Barre syndrome and paralysis than inactivated influenza vaccine (TIV)^11^. Additionally, VAERS reports of intussusception at 1–2 weeks after rotavirus vaccine administration helped to identify this potentially fatal adverse event^43^.

Our study identified 9 AEs associated with all three hepatitis vaccines, of which, 6 AEs (*i.e.*, hepatitis, liver disorder, jaundice, gamma-glutamyltransferase level increased, transaminases level increased, and blood bilirubin level increased) are hepatitis-associated symptoms. It is often difficult to determine whether these AEs are caused by the hepatitis A and/or B vaccines or other factors. Since the three vaccines contain either inactivated hepatitis A virus and/or noninfectious hepatitis B virus surface antigen, it is impossible for the vaccinees to get infections using the vaccinations. However, before the vaccinations, the vaccinees might be exposed to virulent hepatitis A and B viruses, which cause the occurrence of hepatitis and associated symptoms (*e.g.*, transaminases level increased and jaundice). Many vaccinees are the people who have potential risks for exposure to these two viruses. Since the infection of virulent hepatitis A and B viruses have relatively long incubation periods, such infections might not be detected before the vaccinations^32^.

In general, hepatitis A and/or B vaccines are highly safe, as indicated in FDA-approved vaccine package insert documents^37^,^38^,^39^ and peer-reviewed studies^18^,^19^,^20^. However, our studies still found 29 SAEs following the vaccinations using the VAERS data (Tables 1, 2, 3, [Table t2], [Table t3] and Fig. 3). Compared to the spontaneous reported vaccine AE cases in VAERS, the AE records in FDA package inserts were generated from randomized and well-controlled studies and are thus more likely to be causal AEs associated with specific vaccines. The large number of vaccinees reported in VAERS had varied backgrounds (*e.g.*, gender, race, age, and location) and pre-existing health conditions. In contrast, the randomized and well-controlled studies recorded in the package inserts were usually conducted in a small scale of healthy population. Therefore, there are usually less numbers of AEs recorded in FDA package inserts, and the analysis of VAERS data allows the identification of more AEs under special patient backgrounds and conditions. The 29 SAEs identified in our study are enriched in the area of immune system. How these SAEs are related to the hepatitis A and/or B vaccines under various conditions deserves further investigation.

With various data resources and data analysis methods, many groups have identified SAEs associated with hepatitis A vaccines^44^,^45^, hepatitis B vaccines^46^,^47^,^48^,^49^, and hepatitis A and B combination vaccines^32^,^50^ in humans at different ages. Some of these studies also used VAERS data^32^,^44^,^46^,^47^,^48^,^49^. In general, the findings from our study are consistent with previous results^12^,^32^,^46^. Below we focus our discussion on two important areas: autoimmune-related disorder and abortion AEs.

Epidemiological studies and retrospective reviews have shown an association between autoimmunity with hepatitis A and B vaccines^12^,^51^,^52^,^53^,^54^. By evaluating many clinical and laboratory findings of children following hepatitis A vaccination, Karali *et al.* found that none of the children developed autoimmune disorders although hepatitis A vaccine could induce the production of autoantibodies^52^. A case-control epidemiological study described by Geier *et al.* showed that hepatitis B vaccination to adults was associated with an increased risk of serious autoimmune adverse events (SAAEs) such as alopecia, thrombocytopenia, lupus erythematosus, and rheumatoid arthritis^12^. By conducting a nested case-control study within the General Practice Research Database (GPRD) in the United Kingdom, Miguel *et al.* discovered that vaccinees immunized with the hepatitis B vaccine would suffer an increased risk of multiple sclerosis, an autoimmune demyelinating disease^51^. A case report study by Csepregi A *et al.* suggested that Twinrix led to an acute exacerbation of an unrecognized autoimmune hepatitis^53^. In our VAERS study, no autoimmune-related AE associated with hepatitis A vaccine (Havrix) was identified. This result is consistent with the reviewed findings by Karali *et al.*^52^ that people vaccinated with hepatitis A vaccine are unlikely to develop any autoimmune disorders. For hepatitis B and hepatitis AB vaccines, we identified 5 (*i.e.*, multiple sclerosis, myelitis, polyarthritis, rheumatoid arthritis, and systemic lupus erythematosus) and 7 (*i.e.*, autoimmune thyroiditis, ulcerative colitis, multiple sclerosis, myelitis, psoriasis, rheumatoid arthritis, and systemic lupus erythematosus) autoimmune-related AEs associated with Engerix-B and Twinrix, respectively. These findings echoed previous findings of the associations between hepatitis B or AB vaccines and autoimmune diseases^12^,^51^,^53^. However, to date, no clinical evidence exists regarding the causalities between these two types of vaccines and autoimmune diseases. Due to the frequent observations of autoimmune diseases developed after hepatitis B or AB vaccinations, it appears critical to further investigate possible causalities and underlying mechanisms.

Spontaneous abortion (SAB) is the most common pregnancy-specific vaccine AE^55^,^56^,^57^,^58^. From reviewing all VAERS reports for AEs during 1996–2013, Moro *et al.* did not identify any concerning pattern of AEs in pregnant women or their infants following maternal hepatitis A or hepatitis AB immunizations during pregnancy^44^. In our study, out of 1,624 Twinrix case reports, 5 cases included abortion AE (Table 3). Our further VAERS data investigation found that among the 5 cases of abortion following Twinrix vaccination, only 2 cases were spontaneous abortion, and the other 3 were elective termination (VAERS case IDs: 209240, 233067, and 245750). If we only consider the spontaneous abortion and exclude elective termination, the abortion AE would not be classified as significantly associated with Twinrix. In our study, abortion AE and two more specific abortion AEs (*i.e.*, abortion missed and abortion spontaneous) were identified as statistically significant AEs associated with Havrix (Table 1). Specifically, out of 941 Havrix AE case reports, 4, 3, and 29 cases were reported to have abortion, missed abortion (*i.e.*, silent miscarriage), and spontaneous abortion, respectively (Table 1). The rate of abortion (36 over 941 or 3.8%) was considered high and calculated as statistically significant when it was compared with the abortion AE associated with all other vaccines. Such observations suggest that hepatitis A vaccine (Havrix) is more likely to induce abortion-related AE than hepatitis B vaccine (Engerix-B), possibly because Engerix-B is composed of a recombinant protein while Havrix uses the whole inactivated virus. To examine the details about these abortion cases, Supplementary Table S2 was generated to lay out the detailed information about these 36 cases. As shown in this supplementary Table, most (33/36) patients were labeled as coming from “foreign”, and the gestational age at abortion varied from 4 weeks to 38 weeks. It is likely that the patients vaccinated in “foreign” territories were associated with other unexpected effects.

To our best knowledge, our study represents the first report of the analysis of vaccine-vaccine interactions (VVIs) using clinically reported AE case data. Our signal detection algorithm based on a logistic regression model accompanied with MCMC sampling is also the first to be used for detecting potential synergistic VVI or drug-drug interaction (DDI) effects. Many *in silico* models have been developed to predict potential DDIs. For example, a heterogeneous network-assisted inference (HNAI) framework was used to support the prediction of DDIs by integrating drug phenotypic, therapeutic, chemical, and genomic properties^59^. Logistic regression models were also used to predict DDIs using drug clinical AE case report data^60^,^61^. However, these DDI logistic regression models were not accompanied with MCMC sampling (which was used to model fitting)^60^,^61^. Although already reported in statistics^62^,^63^, the logistic regression model with MCMC sampling method is new in VVI and DDI studies. In this work, we first applied the logistic regression model accompanied with MCMC sampling method to estimate the synergistic effect of hepatitis A and B vaccines, which relies on a logistic regression model and makes inference based on the posterior distribution of the fold change (probability of AE for the combination over the summation of two vaccines alone). Our statistical analysis identified 13 significant AEs likely associated with VVIs (Table 4). These AEs were not present or weakly present in Havrix and Engerix-B case reports; however, each of them was strongly associated with the combination vaccine Twinrix. The results suggest that the vaccine contents in Havrix and Engerix-B have significant synergistic interactions which likely result in these 13 AEs in Twinrix-vaccinated patients. Further experimental verifications on these VVI-associated AEs would be important to evaluate the safety of the combinational usage of Havrix and Engerix-B.

## Conclusions

In this paper, we investigated the AEs associated with two monovalent vaccines (*i.e.*, Havrix and Engerix-B) and one combination vaccine (Twinrix) against hepatitis A and B diseases using the data from the VAERS database. The contributions of our study are multiple. First, by using three biometrical methods (PRR, Chi-square test, and base level filtration), we identified 144 unique statistically significant AEs related to the three vaccines. Among these 144 AEs, 29 were considered serious AEs (SAEs). Many SAEs, including autoimmune-related disorder AEs, deserve further investigation. Second, our study is the first time to statistically evaluate the impact of VVI synergistic effects on the AEs using clinically reported AE case data. Such VVI synergistic effects can increase the risk of some special AEs or exacerbate known adverse reactions. To support the VVI study, we developed a statistical method using logistic regression model with MCMC sampling. Thirteen VVI-associated AEs were identified in our study. Third, we compared the AE classification methods using OAE and MedDRA and confirmed the advantages of using OAE for AE classifications. Overall, our research methods and results facilitate vaccine safety surveillance and benefit rational design of more secure and effective vaccines.

## Additional Information

**How to cite this article**: Xie, J. *et al.* Statistical and Ontological Analysis of Adverse Events Associated with Monovalent and Combination Vaccines against Hepatitis A and B Diseases. *Sci. Rep.* **6**, 34318; doi: 10.1038/srep34318 (2016).

## Supplementary Material

Supplementary Information

## Figures and Tables

**Table 1:** Havrix-specific adverse events.

Adverse Event	Count	PRR	Chi-square
Behavioral and neurological AE			
Paraplegia*	4	8.32	24.58
Complex regional pain syndrome	3	6.32	12.95
Impaired work ability	6	2.31	4.39
Oral discomfort	3	6.73	14.10
Paresis	4	2.92	4.96
Pelvic pain	13	14.31	148.59
Polymyalgia rheumatica	3	3.88	6.27
Cardiovascular AE			
Anaphylactic shock*	4	2.71	4.39
Circulatory shock*	6	2.82	6.95
Decreased heart rate	8	3.28	12.47
Hypotension	11	2.16	6.78
Eye AE			
Ocular icterus	3	10.89	25.33
Double vision	7	2.18	4.36
Gustatory system AE			
Anorexia	18	2.14	10.87
Hair, skin or nail AE			
Dry skin	6	2.33	4.49
Hematopoietic system AE			
Eosinophilia	3	4.16	7.04
Hepatobiliary or pancreatic AE			
Jaundice	12	5.33	41.04
Liver disorder	4	4.81	11.73
Homeostasis AE			
Eye edema	4	3.26	6.17
Immune system AE			
Hepatitis A*	13	19.62	206.22
Osteoarthritis*	4	3.94	8.60
Vasculitis*	7	3.22	10.53
Allergic rhinitis	3	2.82	6.95
Hepatitis	8	5.05	25.31
Investigation result abnormal AE			
Alanine aminotransferase level increased	39	5.90	154.61
Aspartate aminotransferase level increased	29	4.42	75.25
Blood bilirubin level increased	10	4.09	22.86
Gamma-glutamyltransferase level increased	15	8.02	88.22
Hepatic enzyme increased	8	5.29	27.03
Transaminase level increased	3	3.18	4.40
Musculoskeletal and connective tissue AE			
Fasciitis	6	4.14	13.99
Fibrosis tendinous	4	6.15	16.65
Myofascitis	7	7.70	39.10
Pregnancy, neonatal or perinatal disorder AE			
Abortion*	4	4.81	11.73
Abortion missed*	3	9.30	21.09
Abortion spontaneous*	29	6.07	119.19
Premature labor*	5	5.43	17.54
Unintended pregnancy	30	17.96	435.41
Reproductive system AE			
Ovarian cyst	3	5.02	9.38
Placental disorder	3	15.99	38.56
Vaginal hemorrhage	26	14.88	310.28
Sexual dysfunctions	3	11.12	25.96
Respiratory system AE			
Painful respiration	3	4.13	6.95
Urinary system AE			
Urinary incontinence*	9	2.46	7.69
Chromaturia	4	4.16	9.38
Nephrotic syndrome	3	5.67	11.22

^*^Serious adverse event (SAE).

**Table 2 t2:** Engerix-B-specific adverse events.

Adverse Event	Count	PRR	Chi-square
Behavioral and neurological AE			
Balance disorder	80	2.55	71.43
Clumsiness	9	2.48	7.52
Depression	43	2.10	23.72
Difficulty in walking	45	3.61	78.38
Disturbance in attention	64	2.99	79.60
Dysgraphia	13	3.45	20.82
Hemiparesis	35	3.29	51.85
Fibromyalgia	35	5.09	102.34
Memory impairment	57	3.90	112.51
Motor dysfunction	29	4.82	78.61
Muscle contractions involuntary	14	2.39	10.66
Paresis	22	4.13	47.45
Vertigo	78	2.31	55.36
Sensory disturbance	69	3.81	131.51
Brain AE			
Cerebellar syndrome	38	17.51	413.55
Digestive system AE			
Tongue disorder	14	2.14	8.12
Ear AE			
Vestibular disorder	11	10.88	77.85
Eye AE			
Double vision	49	3.90	96.90
Dry eye	9	2.37	6.74
Eye disorder	27	2.03	13.44
Nystagmus	22	4.00	45.14
Scotoma	8	6.49	32.03
Visual acuity reduced	50	5.53	163.52
Visual disturbance	48	3.87	93.59
Hepatobiliary or pancreatic AE			
Hepatomegaly*	9	2.57	8.13
Jaundice	20	2.16	11.82
Liver disorder	9	2.66	8.79
Immune system AE			
Hepatitis B*	8	2.15	4.67
Multiple sclerosis*	231	15.77	2312.90
Osteoarthritis*	14	3.48	22.85
Polyarthritis*	16	3.96	32.25
Psoriasis*	15	5.29	46.20
Rheumatoid arthritis*	37	4.18	81.34
Systemic lupus erythematosus*	28	4.14	60.72
Vasculitis*	25	2.87	28.56
Central nervous system inflammation	9	6.19	33.97
Hepatitis	40	6.82	170.24
Multiple sclerosis relapse	8	8.77	45.29
Investigation result abnormal AE			
Blood immunoglobulin A level increased	10	7.80	49.73
Blood bilirubin level increased	22	2.20	13.75
Gamma-glutamyltransferase level increased	20	2.58	18.28
Transaminase level increased	16	4.38	37.76
Metabolism, endocrine or exocrine system AE			
Hypothyroidism	11	3.08	14.35
Musculoskeletal or connective tissue AE			
Fasciitis	82	19.45	971.39
Fibrosis tendinous	44	25.13	629.96
Myofascitis	46	16.08	466.37
Muscular atrophy	38	6.61	155.96
Muscle disorder	34	7.04	150.22
Tendonitis	16	2.28	10.87
Nervous system AE			
Myelitis*	14	3.19	19.54
Carpal tunnel syndrome	10	3.50	16.42
Central nervous system lesion	13	3.18	18.00
Demyelination	90	8.75	509.95
Dysesthesia	10	5.71	34.09
Extrapyramidal disorder	11	18.59	125.56
Formication	16	4.57	40.14
Hyperreflexia	22	4.67	57.00
Myelopathy	8	8.54	44.00
Neuritis	12	2.86	13.60
Neuropathy	26	5.22	78.70
Optic neuritis	46	5.79	160.01
Optic neuritis retrobulbar	20	30.04	322.66
Polyneuropathy	16	3.33	24.10
Pyramidal tract syndrome	14	33.40	241.34
Urinary system AE			
Dysuria	27	3.40	42.31
Micturition disorder	11	20.28	134.43
Proteinuria	10	2.20	6.25
Urgent urination	13	6.06	47.82
Urinary tract obstruction	11	14.87	104.16

^*^Serious adverse event (SAE).

**Table 3 t3:** Twinrix-specific adverse events.

Adverse Event	Count	PRR	Chi-square
Behavioral and neurological AE			
Monoplegia*	5	2.41	4.04
Quadriplegia*	4	3.98	8.57
Facial paresis	11	2.97	14.02
Hypoesthesia facial	12	2.34	9.01
Impaired driving ability	5	3.18	7.22
Impaired work ability	12	2.70	12.50
Monoparesis	4	4.47	10.32
Paraparesis	5	7.03	24.15
Paresis	13	5.69	47.53
Pelvic pain	7	4.28	16.87
Performance status decreased	6	36.91	152.45
Sensory disturbance	24	3.03	31.87
Vertigo	33	2.30	23.82
Cardiovascular AE			
Anaphylactic shock*	8	3.23	11.92
Circulatory collapse*	16	6.91	75.63
Hematoma	9	2.375	7.01
Cardiovascular disorder	13	10.40	99.99
Sinus tachycardia	4	2.68	4.10
Digestive AE			
Eructation	4	4.98	12.13
Feces pale	6	6.95	28.55
Eye AE			
Ocular icterus	4	8.56	24.57
Double vision	14	2.53	12.70
Eye hemorrhage	4	7.29	20.22
Ophthalmoplegia	6	6.42	25.78
Pupils unequal	4	8.20	23.35
Visual disturbance	21	3.90	43.70
Hematopoietic system AE			
Leukopenia	5	2.51	4.44
Splenomegaly	6	4.96	18.08
Hepatobiliary or pancreatic AE			
Hepatic steatosis*	9	2.38	7.01
Hepatomegaly*	5	3.37	8.07
Cholelithiasis	5	9.11	33.06
Cholestasis	4	17.89	53.99
Jaundice	37	10.14	277.12
Liver disorder	10	7.24	50.11
Homeostasis AE			
Laryngeal edema	4	3.90	8.29
Immune system AE			
Autoimmune thyroiditis*	4	5.54	14.11
Hepatitis A*	21	19.68	310.58
Hepatitis B*	6	3.86	12.24
Hepatosplenomegaly*	5	9.28	33.79
Leukocytoclastic vasculitis*	4	4.23	9.48
Multiple sclerosis*	27	3.33	42.79
Psoriasis*	4	3.12	5.60
Rheumatoid arthritis*	9	2.29	6.40
Systemic lupus erythematosus*	8	2.68	8.20
Ulcerative colitis*	4	4.58	10.69
Vasculitis*	10	2.67	10.23
Central nervous system inflammation	4	6.15	16.25
Dermatitis allergic	5	3.39	8.17
Hepatitis	19	7.22	95.01
Lymphocytosis	4	4.80	11.48
Investigation result abnormal AE			
Alanine aminotransferase level increased	73	6.68	332.31
Aspartate aminotransferase level increased	93	8.53	573.00
Blood alkaline phosphatase increased	21	4.90	62.22
Blood bilirubin level increased	30	7.44	155.81
Blood cholesterol increased	5	2.75	5.42
Blood lactate dehydrogenase level increased	16	4.41	40.48
Gamma-glutamyltransferase level increased	59	21.11	932.75
Hepatic enzyme increased	35	14.91	395.11
Monocytosis	4	9.84	28.89
Pleocytosis	6	6.56	26.53
Transaminase level increased	14	9.19	93.49
Musculoskeletal or connective tissue AE			
Arthropathy	8	3.23	11.92
Bone disorder	4	4.47	10.32
Myositis	7	2.46	5.93
Muscle disorder	9	4.01	19.55
Muscular atrophy	7	2.61	6.78
Rhabdomyolysis	4	2.96	5.04
Nervous system AE			
Myelitis*	12	6.56	53.09
Central nervous system lesion	5	2.83	5.75
Demyelination	13	2.59	12.40
Dysesthesia	6	3.69	11.36
Formication	6	3.89	12.38
Optic neuritis	15	4.18	34.91
Polyneuropathy	10	4.90	29.57
Pregnancy, neonatal or perinatal AE			
Abortion*	5	3.49	8.59
Premature delivery*	4	6.56	17.68
Reproductive system AE			
Vaginal hemorrhage	14	4.44	35.83
Respiratory system AE			
Hyperventilation	9	2.11	5.19
Lung DISORDER	7	2.75	7.56
Tumor AE			
Lymphoma*	4	9.84	28.89
Urinary system AE			
Chromaturia	12	7.57	63.61
Proteinuria	6	3.14	8.49

^*^Serious adverse event (SAE).

**Table 4:** VVI analysis results using the logistic regression model with MCMC sampling.

Adverse Event	*p*_*A*_	*p*_*B*_	*p*_*AB*_	*p*_*FC*2_	*p*_*FC*1_
Cardiovascular disorder	0	5.15 × 10^−4^ (2)	8.00 × 10^−3^ (13)	0.999	0
Hepatosplenomegaly	0	0	3.08 × 10^−3^ (5)	0.997	0.001
Premature delivery	0	0	2.46 × 10^−3^ (4)	0.994	0.003
Sinus tachycardia	0	0	2.46 × 10^−3^ (4)	0.994	0.003
Hypoesthesia facial	0	1.54 × 10^−3^ (6)	7.39 × 10^−3^ (12)	0.960	0.001
Lung disorder	0	7.72 × 10^−4^ (3)	4.31 × 10^−3^ (7)	0.950	0.004
Monoparesis	0	2.57 × 10^−4^ (1)	2.46 × 10^−3^ (4)	0.942	0.011
Cholelithiasis	0	5.15 × 10^−4^ (2)	3.08 × 10^−3^ (5)	0.904	0.014
Allergic dermatitis	0	5.15 × 10^−4^ (2)	3.08 × 10^−3^ (5)	0.904	0.014
Feces pale	0	7.72 × 10^−4^ (3)	3.69 × 10^−3^ (6)	0.872	0.012
Pupils unequal	0	5.15 × 10^−4^ (2)	2.46 × 10^−3^ (4)	0.875	0.022
Hepatic steatosis	1.06 × 10^−3^ (1)	5.15 × 10^−4^ (2)	5.54 × 10^−3^ (9)	0.821	0.033
Monoplegia	0	7.72 × 10^−4^ (3)	3.08 × 10^−3^ (5)	0.805	0.041

The number in () represents the number of case reports containing this AE.
