# Supplementary material for: Statistical and Ontological Analysis of Adverse Events Associated with Monovalent and Combination Vaccines against Hepatitis A and B Diseases
Source: Sci Rep. 2016 Oct 3;6:34318. doi: 10.1038/srep34318 (PMC5046117; doi:10.1038/srep34318)
Supplement: Supplementary Information [file srep34318-s1.pdf]

Statistical and Ontological Analysis of Adverse Events Associated with Monovalent and Combination Vaccines against Hepatitis A and B Diseases

Jiangan Xie[1,2], Lili Zhao[3], Shangbo Zhou[1*], Yongqun He[2*]

[1] Key Laboratory of Dependable Service Computing in Cyber Physical Society, Ministry of Education, Chongqing University, Chongqing, China

[2] University of Michigan Medical School, Ann Arbor, Michigan, United States of America

[3] School of Public Health, University of Michigan, Ann Arbor, Michigan, United States of America

[*]Corresponding Authors

Prof. Yongqun He

Email: yongqunh@med.umich.edu

Prof. Shangbo Zhou

Email: shbzhou@cqu.edu.cn

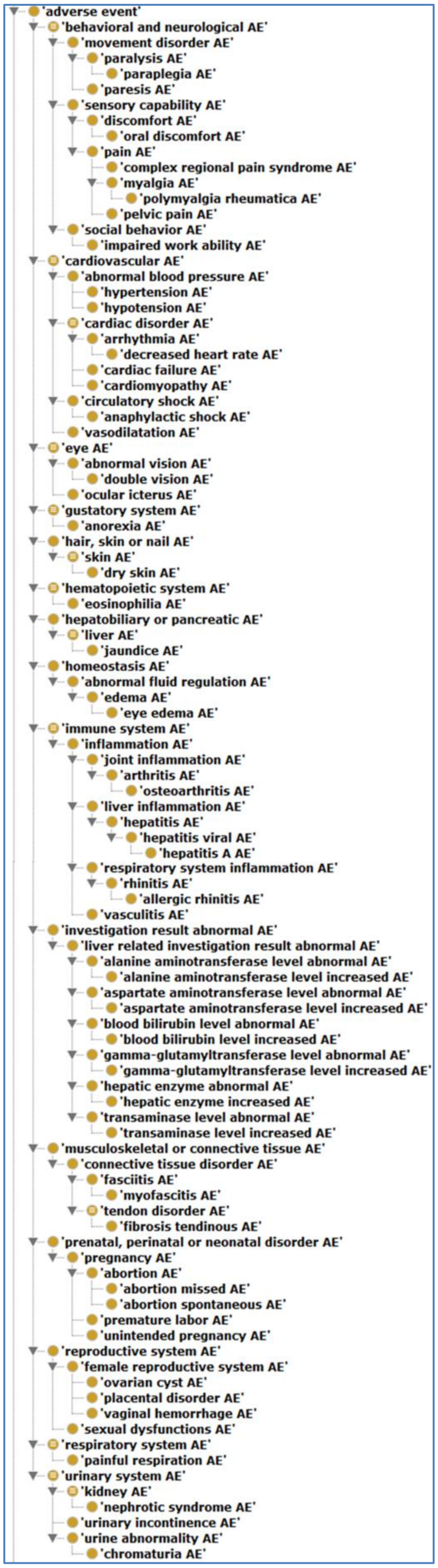

Supplementary Figure S1. Classification of Havrix-specific AEs using OAE.

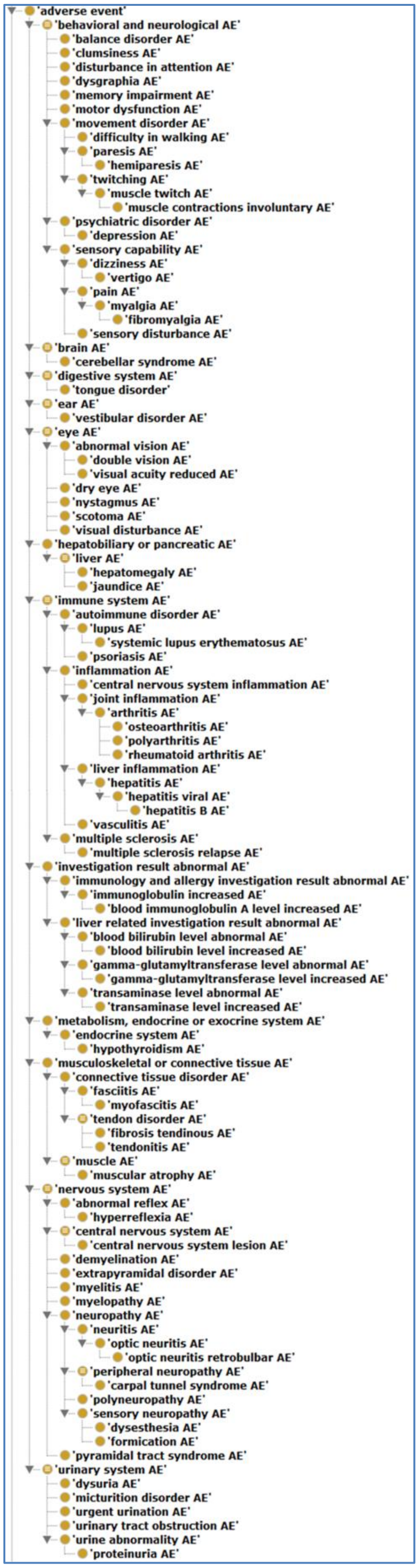

Supplementary Figure S2. Classification of Engerix-B-specific AEs using OAE.

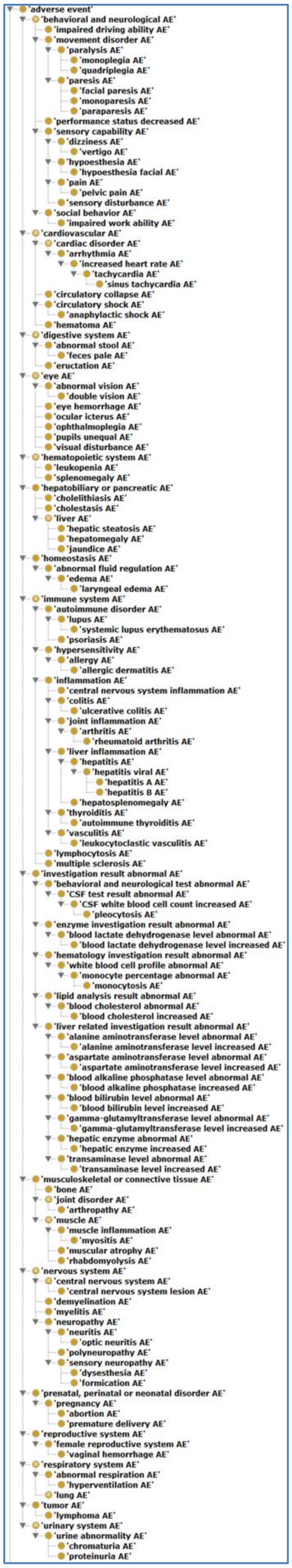

Supplementary Figure S3. Classification of Twinrix-specific AEs using OAE.

Supplementary Table S1. Calculation of PRR and x^2 for vaccine adverse events.

	Vaccine of interest	All other vaccines in VAERS
Adverse event of interest	a	b
All other adverse events	c	d

For the 2×2 contingency table, the PRR and x^2 is calculated by the formula (1) and formula (2), respectively.

$$PRR = \frac{a/(a+c)}{b/(b+d)} \quad (1)$$

$$x^2 = \frac{(ad-bc)^2(a+b+c+d)}{(a+b)(c+d)(b+d)(a+c)} \quad (2)$$

Supplementary Table S2. The detailed information of 36 Havrix-associated abortion-related AEs from VAERS.

VAERS ID	Adverse event	State/Territory	Age (years)	Gestational age when abortion (weeks)
225813	abortion	foreign	36	8
252269	abortion	foreign	26	4
259322	abortion	foreign	22	6
269828	abortion	unknown	18	12
198486	abortion missed	foreign	31	8
288023	abortion missed	foreign	24	20
356348	abortion missed	foreign	25	7.5
228825	abortion spontaneous	foreign	32	38
233578	abortion spontaneous	foreign	26	5
240919	abortion spontaneous	foreign	20	7
251850	abortion spontaneous	foreign	23	9
253346	abortion spontaneous	foreign	22	8
253506	abortion spontaneous	foreign	20	12
253547	abortion spontaneous	foreign	21	12
253741	abortion spontaneous	foreign	26	7
253821	abortion spontaneous	foreign	26	34
254084	abortion spontaneous	foreign	26	14
254085	abortion spontaneous	foreign	25	16.3
254086	abortion spontaneous	foreign	25	unknown
254087	abortion spontaneous	foreign	21	10
254088	abortion spontaneous	foreign	38	8
254089	abortion spontaneous	foreign	19	unknown
254090	abortion spontaneous	foreign	25	unknown
258372	abortion spontaneous	foreign	26	unknown
258951	abortion spontaneous	foreign	19	9.5
259192	abortion spontaneous	unknown	20	13
259991	abortion spontaneous	foreign	21	10
261434	abortion spontaneous	foreign	26	8
262585	abortion spontaneous	foreign	26	8
263087	abortion spontaneous	foreign	21	29.2
266515	abortion spontaneous	foreign	22	20
303071	abortion spontaneous	foreign	30	unknown
334154	abortion spontaneous	foreign	23	unknown
343848	abortion spontaneous	pennsylvania	31	unknown
356348	abortion spontaneous	foreign	25	7.5
449364	abortion spontaneous	foreign	32	unknown
